# Development of an Opsonophagocytic Killing Assay Using HL-60 Cells for Detection of Functional Antibodies against *Streptococcus pyogenes*

**DOI:** 10.1128/mSphere.00617-18

**Published:** 2018-12-19

**Authors:** Sanaz Salehi, Claudia M. Hohn, Thomas A. Penfound, James B. Dale

**Affiliations:** a Department of Medicine, University of Tennessee Health Science Center, Memphis, Tennessee, USA; b Department of Microbiology, Immunology, and Biochemistry, University of Tennessee Health Science Center, Memphis, Tennessee, USA; c Veterans Affairs Medical Center Research Service, Memphis, Tennessee, USA; U.S. Food and Drug Administration

**Keywords:** *Streptococcus pyogenes*, group A streptococcus, opsonic antibodies, vaccines

## Abstract

The clinical development of group A streptococcal (GAS) vaccines will require the implementation of a standardized, high-throughput assay to measure the activity of functional opsonic antibodies in vaccine recipients. In the present study, we adapted and modified the HL-60-based protocol that was developed for the detection of opsonic antibodies against Streptococcus pneumoniae for use with multiple M types of GAS. Modifications of the assay conditions permitted the evaluation of 21 different M types of GAS in the assay. The specificity of the antibody-mediated opsonization was demonstrated by inhibition with homologous, but not heterologous, M proteins. Maximum rates of opsonophagocytic killing (OPK) of 14 different M types promoted by rabbit antiserum against the 30-valent M protein-based vaccine were comparable in whole-blood and HL-60 assays. Data are also presented showing OPK serum titers (opsonic index) of naturally acquired human antibodies present in IVIG [intravenous immune globulin (human)]. Results of the HL-60 assay performed on different days using 21 different M types of GAS and IVIG as the antibody source were significantly concordant. This report indicates that the OPK assay conditions may be optimized for the measurement of opsonic antibodies against a number of epidemiologically important M types of GAS and, once standardized, should facilitate the clinical development of effective vaccines to prevent these infections.

**IMPORTANCE** Measuring functional opsonic antibodies against group A streptococci is an important component of the clinical development path for effective vaccines. Prior studies have used an assay developed over 60 years ago that relied on whole human blood as the source of phagocytes and complement, both of which are critical components of antibody-mediated killing assays. In this study, we adapted an assay that uses the HL-60 human promyelocytic leukemia cell line as phagocytic cells and baby rabbit serum as a source of complement for detection of opsonic antibodies against group A streptococci. On the basis of some of the known biological characteristics of the bacteria, we modified the assay conditions to support the evaluation of 21 epidemiologically important M types and demonstrated the utility and reproducibility of the assay for measurement of functional opsonic antibody levels.

## Abstract (precis)

Measuring functional opsonic antibodies against group A streptococci is an important component of the clinical development path for effective vaccines. Prior studies have used an assay developed over 60 years ago that relied on whole human blood as the source of phagocytes and complement, both of which are critical components of antibody-mediated killing assays. In this study, we adapted an assay that uses the HL-60 human promyelocytic leukemia cell line as phagocytic cells and baby rabbit serum as a source of complement for detection of opsonic antibodies against group A streptococci. On the basis of some of the known biological characteristics of the bacteria, we modified the assay conditions to support the evaluation of 21 epidemiologically important M types and demonstrated the utility and reproducibility of the assay for measurement of functional opsonic antibody levels.

## INTRODUCTION

Group A streptococci (GAS) are ubiquitous human pathogens that cause an estimated 600 million infections worldwide each year (1). The acute infections range from uncomplicated pharyngitis, cellulitis, and pyoderma to life-threatening infections that include necrotizing fasciitis, sepsis, pneumonia, and streptococcal toxic shock syndrome. Mild, even asymptomatic infections can be followed by serious autoimmune diseases, the most significant being acute rheumatic fever (ARF) and rheumatic heart disease (RHD). Although GAS infections are global in their distribution, 95% of the overall burden of GAS infections is found in low- and middle-income countries of the world. The Global Burden of Disease (GBD) study estimated that RHD affects 34,000,000 worldwide, with more than 345,000 deaths per year (2). Invasive disease results in an additional 160,000 deaths per year (1). RHD has contributed to disease burdens totaling over 10,000,000 disability-adjusted life years (DALYs) (3). The combination of the mortality rates associated with rheumatic heart disease (RHD) and invasive GAS infections represents the fifth leading cause of single-pathogen infectious disease deaths, behind HIV, tuberculosis, malaria, and Streptococcus pneumoniae (1, 2).

The massive global burden of disease associated with GAS infections has been the impetus for vaccine development that has spanned many decades (4, 5). Previously, we studied the functional immunogenicity of several multivalent M protein-based vaccines, including 6-valent, 26-valent, and, more recently, 30-valent constructs (6–11). The M protein of GAS has been considered a promising vaccine candidate because M antibodies protect animals against challenge infections (12). While there is not an established immune correlate of protection in humans, opsonic antibodies directed against the M protein that mediate phagocytic uptake and killing have been associated with protection against symptomatic pharyngitis (13, 14). The detection of functional opsonic M protein antibodies described by Lancefield as the “indirect bactericidal assay” (15) entailed the use of “nonimmune” human blood as a source of complement and phagocytes. The classical Lancefield assay is cumbersome and requires fresh human blood that had been prescreened to determine if it will support the growth of the specific GAS M type being tested. The fact that GAS infections are quite common and that most adults have antibodies against multiple M proteins has led to problems with reproducibility and variable results among laboratories. In addition, the complexity of the current 30-valent vaccine, which was designed to elicit opsonic antibodies against 30 different M types of GAS, required screening a number of blood donors to identify nonimmune subjects (10).

The problems outlined above are similar to those encountered during the development of vaccines against Streptococcus pneumoniae. Protection against infection by the pneumococcus is largely mediated by opsonic anticapsular antibodies that mediate phagocytic killing. Development of pneumococcal vaccines has been facilitated by the development of standardized opsonophagocytic killing (OPK) assays using differentiated HL-60 cells to assess functional antibody levels in preimmune and immune serum samples (16, 17). In a recent report, Jones et al. described a protocol adapting the HL-60-based assay for detecting human and rabbit bactericidal antibodies using three different M types of GAS (18). In the current study, we investigated modifications of the assay conditions with the overall goal of expanding the number of M types that may be evaluated using the assay.

## RESULTS

### Optimizing the growth of GAS strains in the presence of active complement and HL-60 cells.

The virulence of GAS in nonimmune hosts is determined in part by their ability to prevent activation and deposition of C3b, the primary opsonin (19). The mechanisms by which this is accomplished depend on the specific M type but include the binding of fibrinogen and IgG to bacterial surface proteins (19, 20). The aim of these initial experiments was to identify components of the OPK assay reaction mixture that resulted in maximum growth of GAS in the presence of phagocytic HL-60 cells and active baby rabbit complement in the absence of specific opsonic antibodies, thereby reducing nonspecific killing of the test bacteria and maximizing the specificity of the assay for the detection of bactericidal antibodies.

**FIG 1:**
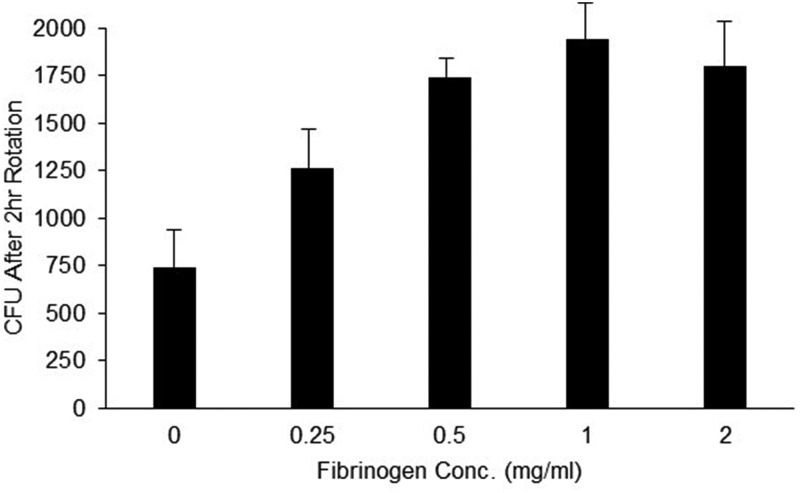
Fibrinogen concentration versus growth of M12 GAS in assay mixtures containing HL-60 cells and active complement. Human fibrinogen was added at the indicated concentrations to the opsonization buffer, which also contained 10 U/ml heparin. The inoculum of M12 GAS was 35 CFU. After rotation at 37°C for 2 h, the colonies surviving were enumerated (error bars indicate standard deviations [SD] of results from four replicate samples).

The addition of fibrinogen in various concentrations to the reaction buffer increased the growth rate of M12 GAS when active complement was present (Fig. 1). Similar results were obtained with M5 and M118 GAS (data not shown). However, when a number of M types (M2, M28, M4, M11, M22, M73, M77, M82, M87, and M89) were rotated in mixtures with and without fibrinogen, there was not a significant difference in survival rates of the bacteria in reaction mixtures containing HL-60 cells and either inactive or active complement.

Previous studies have shown that many M types of GAS bind human IgG via the Fc receptor to M and/or M-related protein (Mrp) on their surfaces and that this contributes to resistance to phagocytosis (19, 21). IgG binding to the surface Mrp was limited to human and pig IgG, among multiple species tested (22). Therefore, we incubated M22 GAS in HL-60 rotation mixtures that contained increasing concentrations of pig serum as well as a final concentration of 0.5 mg/ml of fibrinogen. In this case, maximum growth of the bacteria in the presence of active complement compared to buffer with heat-inactivated complement was achieved with a final concentration of 12.5% pig serum (data not shown). Similar results were obtained with M77 and M87 GAS.

**FIG 2:**
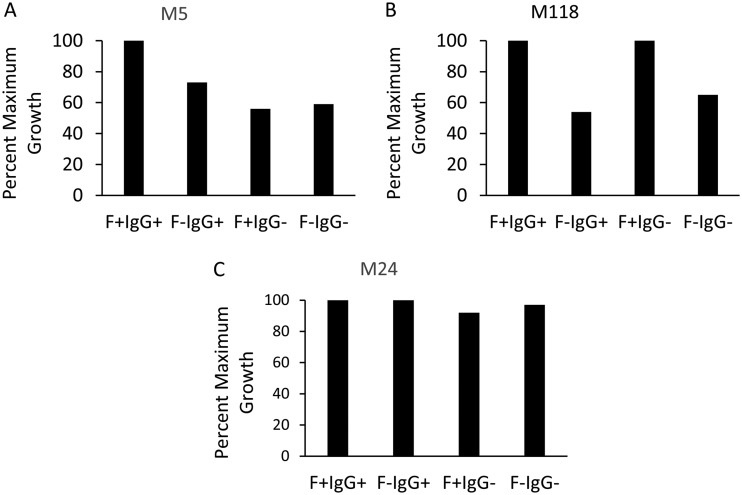
Fibrinogen (F; 0.5 mg/ml) and/or pig serum (IgG; 12.5%) added to the HL-60 reaction mixture had different effects on maximum growth of GAS, depending on the M type. Percent maximum growth was defined as the number of CFU recovered from quadruplicate reaction mixtures containing fibrinogen and pig serum plus HL-60 cells and active complement. (A) M5 required fibrinogen and pig serum to achieve maximum growth. (B) M118 required only fibrinogen to achieve maximum growth. (C) M24 did not require either component to achieve maximum growth.

Our goal was to formulate a universal buffer that could be employed for the majority of M types tested. Not all M types required fibrinogen and/or pig serum for optimal growth (Fig. 2). For example, M5 demonstrated maximum growth in the presence of active complement and HL-60 cells when both fibrinogen and pig serum were present in the reaction mixture (Fig. 2A). The addition of fibrinogen resulted in maximum growth of M118 GAS, but pig serum had no effect (Fig. 2B). Neither fibrinogen nor pig serum was required for optimal growth of M24 (Fig. 2C).

**FIG 3:**
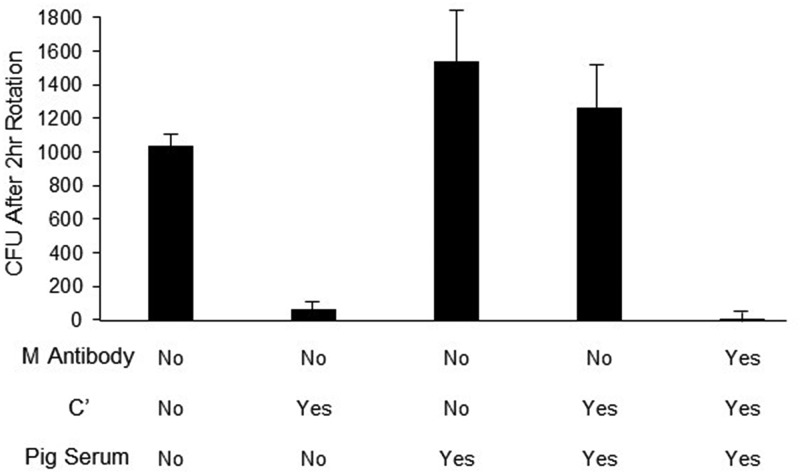
Pig serum in the reaction buffer reduced nonspecific complement-mediated killing of M77 GAS. Quadruplicate wells containing the indicated components were inoculated with 53 CFU of M77 GAS and rotated for 2 h. In the presence of active complement, the addition of pig serum increased the level of the bacteria from 65 CFU (bar 2) to 1,250 CFU (bar 4). The reduction of nonspecific killing permitted the assessment of antibody-mediated killing (bar 5). Error bars represent ±SD of results from quadruplicate samples.

Reduction of nonspecific killing of GAS in the presence of HL-60 cells and complement was a prerequisite for optimizing the assessment of functional OPK activity mediated by vaccine antibodies. To demonstrate this directly, we performed OPK assays with M77 GAS in mixtures that contained fibrinogen but were formulated with and without complement, pig serum, and M antibody (Fig. 3). The bacteria failed to grow in the presence of active complement when no pig serum was present. The addition of pig serum resulted in substantially increased growth, which was completely inhibited when M antibody was included in the mixture. Thus, optimal resistance to nonspecific opsonization by complement permitted the assessment of phagocytic killing mediated by functional M antibodies.

### Optimizing HL-60 cell number and incubation times.

OPK assays were performed with increasing numbers of HL-60 cells (10^4^, 10^5^, and 10^6^/well) and M5 GAS (inoculum of 20 CFU) with 30-valent vaccine rabbit antiserum. Maximum killing was observed with 10^6^ cells/well when M antibodies were included in the reaction mixture (data not shown). The optimal number of HL-60 cells per ml approximates the number of neutrophils in normal human blood. We next incubated the reaction mixtures for 1, 2, or 3 h and determined growth of the inoculum of M5 GAS in the control serum and percent killing mediated by rabbit antisera against the 30-valent vaccine (data not shown). After 1 h, there was insufficient growth of the bacteria to reliably assess killing mediated by M antibodies. A 3-h incubation period resulted in too many CFU in the control wells such that percent killing could not be determined using undiluted samples. Incubation for 2 h resulted in growth of the inoculum to >4 generations and optimal killing in the presence of M antibody. All subsequent experiments were performed using a 2-h incubation period.

### Specificity of the OPK activity mediated by 30-valent vaccine antiserum.

Inhibition assays were used to assess the specificity of the bacterial killing observed in the HL-60 assay. Because the 30-valent vaccine evoked antibodies that cross-opsonized a number of GAS M types (10), we selected M1 and M5 GAS and heterologous recombinant M proteins as inhibitors that, for the most part, contain type-specific M epitopes. The OPK activity of the 30-valent antiserum against M5 GAS was inhibited 100% after preincubation with recombinant M5 (rM5) and inhibited 5% by heterologous rM3 (Table 1). Likewise, the OPK activity against M1 GAS was inhibited 100% after preincubation with rM1 peptide but not at all by rM5. Taken together, these results indicate the M protein specificity of the OPK activity and that the reduction in CFU is mediated by M antibodies directed against opsonic epitopes of the respective M proteins.

**TABLE 1:** Specificity of OPK activity of 30-valent rabbit antiserum as determined by recombinant M protein inhibition assays^*a*^

Strain of test bacteria	Maximum percent killing (% inhibition) after incubation with:		
	No inhibitor	Homologous M	Heterologous M
M5	59	0 (100)	56 (5)^*b*^
M1	86	0 (100)	100 (0)^*c*^

a Inhibition assays were performed in quadruplicate using the same concentration of antiserum in each reaction mixture. The final dilution of rabbit antiserum was 1:8 for M5 bacteria, and the final dilution was 1:16 for M1 bacteria.

b The heterologous antigen was 10 µg rM3, which had been preincubated with the rabbit antiserum prior to addition to the reaction mixture.

c The heterologous antigen was 10 µg rM5.

### OPK activity in whole human blood versus HL-60 cells.

**FIG 4:**
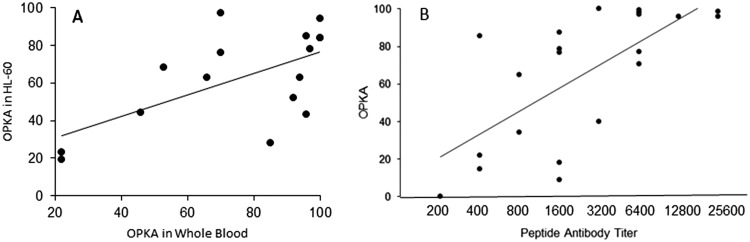
(A) Correlation between OPK activity (OPKA) (maximum killing) levels in whole human blood versus HL-60 cells. (B) Correlation between homologous peptide antibody titer and OPKA. (A) Paired assays were performed simultaneously in either whole human blood or HL-60 cells with the same pool of 30-valent vaccine rabbit antiserum. A total of 16 paired assays with 14 different M types (M1, M2, M3, M4, M5, M6, M12, M19, M24, M44, M58, M82, M89, and M118) were performed (*r* = 0.62, *P* < 0.01 [Pearson correlation coefficient]). (B) OPK activity (maximum killing) with HL-60 cells against M4, M58, M82, M87, and M89 GAS versus titer of 30-valent vaccine antisera against the homologous M peptide. Rabbit antisera with a range of titers against the respective M peptides were used at a 1:4 dilution in the OPK assay (*r* = 0.69, *P* < 0.01 [Pearson correlation coefficient]).

To assess the correlation between the results of the standard Lancefield whole-blood indirect bactericidal assay and the HL-60 OPK assay, a total of 16 paired assays were performed with 14 different M types in the presence of rabbit antisera against the 30-valent vaccine (Fig. 4A). The results indicated a correlation between the maximum killing rates in the two assays (*r* = 0.62, *P* < 0.01). The HL-60 assays appeared to be more sensitive in some cases, but the whole-blood assay showed greater activity in others.

### Relationship of peptide antibody titer to OPK activity.

To assess the relationship between M peptide antibody titer and OPK activity, 30-valent vaccine rabbit antisera with a range of titers against individual N-terminal synthetic peptides, as determined by enzyme-linked immunosorbent assay (ELISA) (8, 10), were assessed for OPK activity against five different M types of GAS (Fig. 4B). There was a significant correlation between peptide-specific titer and maximum killing (*r* = 0.69, *P* < 0.01) across a range of antibody levels.

### Performance of multiple M types of GAS in OPK assays.

The sensitivity and specificity of the OPK assay are directly related to the ability of the GAS strain to resist opsonization by complement and phagocytic killing. Therefore, preliminary acceptance criteria for each assay were established that included (i) growth of the test bacteria to at least four generations as determined by comparing the CFU in the inoculum to the total CFU recovered from control wells containing active complement after the 2-h incubation and (ii) <30% nonspecific killing in the presence of active complement versus heat-inactivated complement. We tested a total of 66 laboratory strains of GAS representing 25 M types and analyzed the results using these criteria. Fourteen of the 66 assays were rejected based on less than four generations of growth of the test bacteria having occurred. Of the remaining 52 assays involving 22 M types, 4 were rejected because nonspecific killing exceeded 30%, leaving 48 assays with 21 different M types. The mean maximum level of OPK activity of 30-valent vaccine rabbit antisera at a dilution of 1:4 in representative assays with 21 M types of GAS was 66% (median, 74%; range, 23% to 98%), and the mean nonspecific killing rate was 10% (median, 8%; range, 0% to 29%) (Table 2).

**TABLE 2:** HL-60 OPK (maximum killing) with 30-valent vaccine rabbit antisera against a panel of group A streptococcal M types

M type	Nonspecific killing (%)	Specific killing (%)
1	0	94
2	8	75
3	8	63
4	0	62
5	0	85
6	0	84
12	18	84
14	6	35
18	0	84
19	0	43
22	8	28
24	0	28
44	20	23
58	18	85
75	23	74
77	19	98
82	0	70
87	28	82
89	0	98
114	24	44
118	29	44
Mean	10.0	66
Median	8	74

### OPK titers of naturally acquired opsonic antibodies in IVIG.

**FIG 5:**
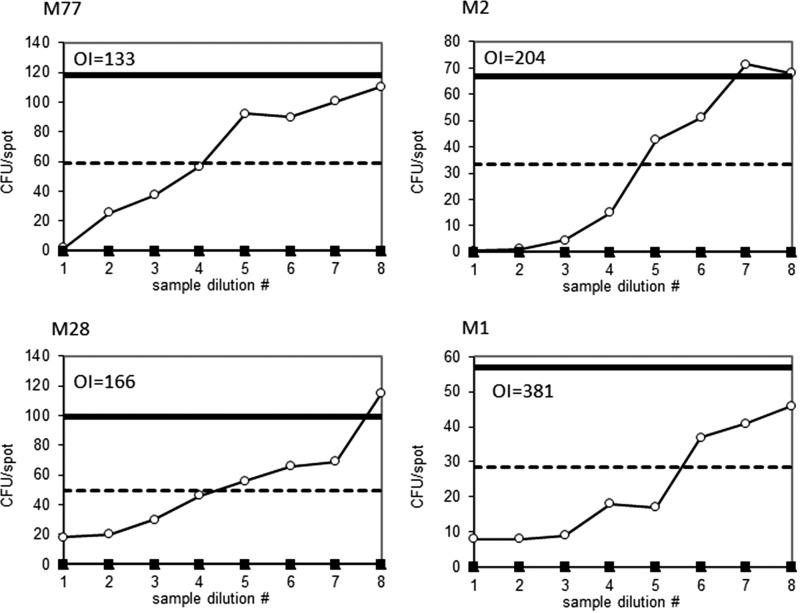
Representative titration curves generated using four M types of GAS incubated with serial 2-fold dilutions of IVIG to determine the opsonic index (OI). The initial dilution of IVIG in each experiment was 1:4. The dashed line in each panel represents 50% killing, and the solid line represents 0% killing. The graphics and the OI were automatically generated using Opsotiter 3.0 software provided by the University of Alabama.

In the experiments described above, OPK activity was defined as the maximum percentage of killing of GAS in mixtures containing opsonic antibodies (final dilution 1:4) compared to control mixtures containing active complement and normal rabbit serum. Quantitative comparisons of opsonic activity may be obtained using serial dilutions of the test antisera and calculating the OPK titer (opsonic index [OI]) defined as the interpolated dilution of antiserum resulting in 50% killing, using the Opsotiter 3 software (16, 23). We determined the opsonic index for IVIG [intravenous immune globulin (human)] using 20 different M types of GAS. As expected, the OI values were highly variable depending on the M type (range, 2 to 4,096; mean, 238). Antibodies against the relatively common M types M77, M2, M28, and M1 produced typical dose response curves and OI values ranging from 133 to 381 (Fig. 5).

### Reproducibility of OPK titers.

**FIG 6:**
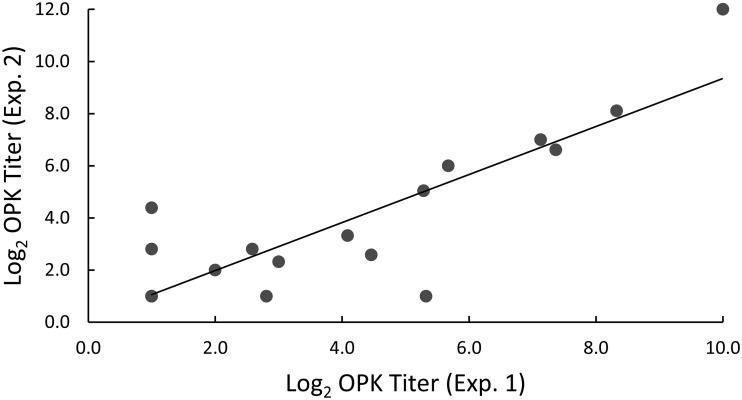
Reproducibility of HL-60 OPK assay with IVIG and multiple different M types of GAS. OPK titers (OI values) were determined on different days using 21 M types of GAS (M1, M2, M3, M4, M5, M6, M12, M14, M18, M19, M22, M24, M28, M44, M75, M77, M82, M87, M89, M114, and M118) incubated with 2-fold dilutions of IVIG starting at 1:4. OI was calculated using Opsotiter 3.0 software (*r* = 0.87, *P* < 0.01 [Pearson correlation coefficient]).

To determine the day-to-day variability of the HL-60-based assay, we determined OPK titers of IVIG against 21 different M types of GAS at different times (Fig. 6). As described above, the IVIG demonstrated a range of bactericidal titers, depending on the M type used in the assay. The overall correlation between the results from the two experiments was significant (*r* = 0.87, *P* < 0.01), and the most consistent results were observed with the M types that resulted in the highest OPK titers.

## DISCUSSION

There is a significant need for a standardized, reproducible functional assay to detect bactericidal antibodies against GAS (18, 24, 25). Once standardized, the assay will be a critical component of vaccine development strategies and may contribute to the establishment of an immune correlate of protection in humans (26). A recent report described OPK assay conditions for three epidemiologically important GAS M types (*emm*1, *emm*6, and *emm*12) using modifications of protocols that have long been established for S. pneumoniae (18). The overall goal of the present study was to extend these observations by assessing additional assay conditions that would permit the evaluation of a broader range of different GAS M types representing many of those included in the current multivalent vaccine and those more likely to be encountered in different geographic locations.

Group A streptococci use a number of different mechanisms to resist opsonization and phagocytosis. As a result, they represent a somewhat heterogenous population of bacteria relative to expression of different surface proteins and binding of specific human plasma proteins that mediate virulence (19). In the present study, we modified the assay conditions described in a previous study for use with S. pneumoniae (16) and in a recent report for GAS (18) based on some of the known virulence characteristics of the bacteria. Modifying the reaction buffer of the OPK assay by adding fibrinogen and pig serum expanded the total number of GAS M types that could be assessed using this technique. The inoculum used in the current assays was from freshly grown log-phase bacteria, as opposed to the use of frozen stocks or late-log-phase cultures. This ensured that the GAS expressed high levels of surface proteins and capsule, both of which may be necessary for some M types to display optimal resistance to phagocytosis in the presence of active complement. In some cases, the bacteria required serial passage in nonimmune human blood (12, 15) or in HL-60 cells plus complement to select for bacteria that were fully resistant to phagocytic killing. The passaged cultures were frozen for future use. To allow growth during the 2-h incubation in the absence of opsonic antibodies and to enhance detection of antibody-mediated killing by HL-60 cells, fewer bacteria were added to the test mixtures than had been used in previously reported studies. These assay conditions were aimed at optimizing the survival of GAS in test mixtures containing fully active complement and HL-60 cells; as such, there was no need to reduce complement activity prior to use in the assay such as had been described in previous studies (18). Because C3b is the primary opsonin that promotes phagocytic uptake of GAS, we reasoned that the use of fully active complement would result in increased sensitivity of the assay.

The protocol described in this report mimics some conditions of the classic whole-blood Lancefield assay by using a small inoculum containing bacteria grown to the early log phase, phagocytic cell numbers that approach those found in human blood, an incubation period sufficient to permit growth of bacteria in the control wells, and end-over-end rotation of the plate to facilitate optimal interaction of the phagocytes with opsonized bacteria. However, the protocol also included a 96-well format with replicate samples and serial dilutions of test serum, which resulted in a high-throughput, reproducible assay with multiple serum samples and different M types of GAS. Automated counting of GAS colonies within hemolytic zones on blood agar plates obviated the need to use 2,3,5-tetraphenyltetrazolium chloride as an indicator dye in an overlay agar.

Although there was general agreement between the results of the whole-blood and HL-60 assays, some results were discordant for some M types tested. This might have been the result of the presence of low levels of preexisting antibodies in the human blood that are not sufficient to opsonize the bacteria in the control samples; however, they might have amplified the OPK activity when vaccine antisera were added. In addition, human serum contains complement regulatory proteins, such as C4 binding protein and factor H, which are known to bind to the N-terminal regions of M protein and to contribute to resistance to opsonization by complement (20, 27). In future studies, it may be necessary to add these purified proteins to optimize the assay conditions for some M types of GAS. Human blood also contains monocytes that may participate in the phagocytic uptake and killing of GAS that are opsonized by M antibodies. Some M types may be more susceptible to killing by neutrophils and monocytes; in such cases, the HL-60 cells could be differentiated into the two phenotypes and then mixed prior to the assay (28).

In this report, we did not attempt to develop a standardized protocol for all GAS M types tested in the HL-60 OPK assay. Rather, we have shown that certain modifications of the assay permitted an evaluation of multiple M types that rely on different mechanisms to resist phagocytic killing. This is an important next step, given the diversity of M types found in the low- and middle-income countries (29) where the burden of disease is greatest (1–3). Further work will be necessary to standardize the assay and to qualify the conditions for each M type, including the development of a harmonized protocol and determination of concordance of results between laboratories (23). Another important step in the development process will be the use of clinical samples from the recently completed phase 1 trial of the 30-valent vaccine (unpublished data) to bridge results from the classical whole-blood assay to those obtained in the HL-60 assay. Eventually, the standardized assay may lead to a correlate of protection when used as a component of vaccine efficacy trials (26).

## MATERIALS AND METHODS

### Bacterial strains.

A total of 25 different M types of group A streptococci were used in these studies. All isolates were from our laboratory collection and represented 30-valent GAS vaccine types that have previously been reported (10). Bacterial strains were stored at −80°C, cultured overnight at 30°C in Todd-Hewitt yeast broth (THYB) containing Todd-Hewitt broth (THB; BD, Sparks, MD) and 1% yeast extract (Bacto yeast extract; BD) with the addition of 7.5% normal rabbit serum (Sigma-Aldrich, St. Louis, MO), and then subcultured into fresh THBY until the optical density at 530 nm (OD_530_) reached 0.08 to 0.1. The log-phase growth cultures were diluted 10^−4^ in fresh THB and placed on ice until they were added to the assay mixtures. GAS M types that showed unacceptable (>30%) levels of nonspecific killing were passaged in whole nonimmune human blood or in the HL-60 assay mixture containing active complement for 2 h and dripped onto blood agar (tryptic soy blood agar base no. 2; BD) plates containing 5% sheep’s blood (Edge Biologicals, Inc., Memphis, TN). THYB was inoculated with single colonies, and overnight cultures were stored at −80°C.

### Antisera.

OPK assays were performed with IVIG [Immune Globulin Intravenous (Human); Gammagard Liquid, 10%] (Baxter Healthcare Corp., Westlake Village, CA) or rabbit antisera against the 30-valent M protein-based vaccine (10). Female New Zealand White rabbits were immunized intramuscularly (i.m.) with three 600-µg doses of vaccine on 1 mg of alum (Rehydragel LV; General Chemical, Berkeley Heights, NJ) at 4-week intervals. Some antisera were prepared using the same dose of vaccine administered subcutaneously in complete Freund’s adjuvant for the first dose and in incomplete adjuvant (catalog. no. 5881 and 5506; Sigma-Aldrich) for the final two doses. Serum was obtained 2 and 4 weeks after the final injection.

### HL-60 cells.

HL-60 cells (CCL-240; ATCC, Manassas, VA) were propagated in RPMI 1640 (Gibco, Grand Island, NY) containing 10% fetal bovine serum (FBS) (FetalClone I; HyClone, Fisher Scientific, Logan, UT) supplemented with 1% l-glutamine (GlutaMAX; Gibco, Life Technologies, MD), 100 U/ml penicillin, and 100 μg/ml streptomycin. The cells were differentiated into neutrophils in RPMI medium without antibiotics but containing 10% fetal calf serum, glutamine, and 0.8% dimethylformamide (DMF; Fisher Scientific) for 5 to 6 days. Prior to use in the assay, the cells were washed in RPMI medium without phenol red and viable cells were counted based on trypan blue exclusion in a hemocytometer. HL-60 cells were resuspended in the opsonization buffer at a concentration of 10^7^ cells/ml. Differentiation into the neutrophil lineage was assessed by flow cytometry using mouse anti-human CD35 and CD71 (BD Pharmingen, San Diego, CA) to confirm that ≥55% of the cells were CD35^+^ and ≤20% of the total cells were CD71^+^ (observed values ranged from 73.5% to 86% and from 0.6% to 4.6%, respectively).

### OPK assay conditions.

The OPK assays were performed with multiple variables with respect to the formulation of the opsonization buffer, the number of HL-60 cells per well, and the time of incubation. The final modified opsonization buffer contained Hanks’ balanced salt solution (HBSS; Corning, Manassas, VA) with Ca/Mg, 0.1% gelatin (Sigma-Aldrich), 25% pig serum (Sigma-Aldrich), 1 mg/ml purified human fibrinogen (Hyphen Biomed, West Chester, OH), and 10 U/ml heparin (Sky, Concord, NC). The choice of pig serum in the reaction mixtures was guided by results of previous studies performed in our laboratory showing that IgG binding to the surface Mrp, which is expressed by the majority of GAS M types, was limited to human and pig IgG, among multiple species tested (22). In certain experiments, the concentrations of the pig serum and fibrinogen were adjusted to determine the optimal conditions for different M types of GAS.

The OPK assay was performed in round-bottom 96-well plates (Corning, Inc., Corning NY). Experiments to determine maximum killing were performed in quadruplicate. OPK titers were determined using 50-µl volumes of eight serial dilutions of the test serum in duplicate. Control wells contained 50 µl of normal rabbit serum (Sigma-Aldrich), 10 µl bacteria, 100 µl HL-60 cells in opsonization buffer, and 40 µl of either heat-inactivated (complement-negative control) or freshly thawed (complement-positive control) baby rabbit serum (PelFreeze Biologicals, Rogers, AR). The bacterial inoculum for each assay was determined by dripping 5 µl of the suspension onto blood agar, as described below. The inoculum ranged from 20 to 100 CFU/well. Bacteria were added to test serum samples and incubated at room temperature for 15 min, after which the opsonization buffer containing HL-60 cells and baby rabbit serum was added. In experiments designed to determine the specificity of the antibody activity, the antisera were preincubated with either homologous or heterologous purified recombinant M antigens copying approximately the N-terminal half of the mature M proteins, previously referred to as “pep M” (6). The plates were sealed with aluminum film (AlumaSeal II AF-100; Excel Scientific, Victorville, CA), attached to a rotator, and incubated with end-over-end rotation at 8 rpm for 2 h at 37°C. The plates were maintained at 4°C for 15 min to stop the reaction. A multichannel pipette was used to pierce the foil, and, after mixing of each well was performed, 5-µl volumes were applied to blood agar plates (Greiner Bio-One, Monroe, NC) (12 by 12 cm square), which were immediately tilted to create a “drip” to spread the bacteria into an ∼0.5-by-3-cm area. The plates were incubated overnight at 37°C.

Surviving bacteria were enumerated using an automated colony counter (ProtoCol3; Synbiosis USA, Frederick, MD). Percent maximum killing was calculated using the average number of CFU surviving the rotation in HL-60 cells with test antiserum plus active complement as a percentage of the CFU in the same mixture without antibodies. Nonspecific killing was calculated using the average CFU recovered in wells containing active complement as a percentage of the CFU surviving in heat-inactivated complement. Generations of growth of the bacteria were calculated by comparing the total CFU (CFU counted × 20) surviving in the active complement control wells (maximum growth) to the CFU contained in the inoculum. Assays were considered acceptable when the proportion of nonspecific killing was <30%, the number of generations of the bacteria grown in the control wells containing active complement was ≥4, and the number of colonies per spot did not exceed 150. OPK titers (OI values) were derived using Opsotiter 3 software (license granted to the licensee by the University of Alabama at Birmingham Research Foundation [UABRF]), and the titers were defined as representing the inverse of the interpolated serum dilution level that resulted in 50% killing of the test bacteria.
